# UV-C Seed Surface Sterilization and Fe, Zn, Mg, Cr Biofortification of Wheat Sprouts as an Effective Strategy of Bioelement Supplementation

**DOI:** 10.3390/ijms241210367

**Published:** 2023-06-20

**Authors:** Katarzyna Czarnek, Małgorzata Tatarczak-Michalewska, Piotr Dreher, Vishnu D. Rajput, Grzegorz Wójcik, Anna Gierut-Kot, Agnieszka Szopa, Eliza Blicharska

**Affiliations:** 1 Institute of Medical Science, Faculty of Medical, The John Paul II Catholic University of Lublin, Konstantynów 1 H Str., 20-708 Lublin, Poland; 2 Department of Pathobiochemistry and Interdisciplinary Applications of Ion Chromatography, Biomedical Sciences, Medical University of Lublin, 1 Chodźki Str., 20-093 Lublin, Poland; malgorzatatatarczakmichalewska@umlub.pl; 3 Chair and Department of Public Health, Medical University of Lublin, 1 Chodźki Str., 20-093 Lublin, Poland; piotr.dreher@umlub.pl; 4 Academy of Biology and Biotechnology, Southern Federal University, 344090 Rostov-on-Don, Russia; rajput.vishnu@gmail.com; 5 Department of Inorganic Chemistry, Institute of Chemical Sciences, Faculty of Chemistry, Maria Curie-Skłodowska University, 20-031 Lublin, Poland; g.wojcik@poczta.umcs.lublin.pl; 6 Intermag sp. z o.o. R+D Department, Al. 1000-Lecia 15G, 32-300 Olkusz, Poland; el2000gato@gmail.com; 7 Chair and Department of Pharmaceutical Botany, Jagiellonian University Medical College, Medyczna 9 Str., 30-688 Kraków, Poland; a.szopa@uj.edu.pl

**Keywords:** iron, zinc, magnesium, chromium, trace elements, SEM, biofortification, atomic absorption spectrometry, wheat sprouts, UV seed sterilization, nutrient deficiency

## Abstract

Metalloenzymes play an important role in the regulation of many biological functions. An effective way to prevent deficiencies of essential minerals in human diets is the biofortification of plant materials. The process of enriching crop sprouts under hydroponic conditions is the easiest and cheapest to conduct and control. In this study, the sprouts of the wheat (*Triticum aestivum* L.) varieties Arkadia and Tonacja underwent biofortification with Fe, Zn, Mg, and Cr solutions in hydroponic media at four concentrations (0, 50, 100, and 200 µg g^−1^) over four and seven days. Moreover, this study is the first to combine sprout biofortification with UV-C (λ = 254 nm) radiation treatment for seed surface sterilization. The results showed that UV-C radiation was effective in suppressing seed germination contamination by microorganisms. The seed germination energy was slightly affected by UV-C radiation but remained at a high level (79–95%). The influence of this non-chemical sterilization process on seeds was tested in an innovative manner using a scanning electron microscope (SEM) and EXAKT thin-section cutting. The applied sterilization process reduced neither the growth and development of sprouts nor nutrient bioassimilation. In general, wheat sprouts easily accumulate Fe, Zn, Mg, and Cr during the applied growth period. A very strong correlation between the ion concentration in the media and microelement assimilation in the plant tissues (R^2^ > 0.9) was detected. The results of the quantitative ion assays performed with atomic absorption spectrometry (AAS) using the flame atomization method were correlated with the morphological evaluation of sprouts in order to determine the optimum concentration of individual elements in the hydroponic solution. The best conditions were indicated for 7-day cultivation in 100 µg g^−1^ of solutions with Fe (218% and 322% better nutrient accumulation in comparison to the control condition) and Zn (19 and 29 times richer in zinc concentration compared to the sprouts without supplementation). The maximum plant product biofortification with magnesium did not exceed 40% in intensity compared to the control sample. The best-developed sprouts were grown in the solution with 50 µg g^−1^ of Cr. In contrast, the concentration of 200 µg g^−1^ was clearly toxic to the wheat sprouts.

## 1. Introduction

Trace elements function in the human body mainly as catalysts for cellular enzymes to perform various functions, the most important of which is their structural role in specific metalloenzymes. These metals remain a constant component of such enzyme complexes, and their amount depends on the structure of the enzyme involved. The specificity of enzymatic reactions often requires the use of specific ions by the complex, which cannot be exchanged for other ions or will have an inhibitory effect on a given enzymatic reaction [1,2].

Iron is the most abundant metal in the human body. Body Fe content is approximately 3–4 g, which corresponds to a concentration of 40–50 mg of Fe per kilogram of body weight [3]. In the human body, this element occurs in the form of complexes with proteins (hemoproteins), among others, as a component of heme, heme enzymes, and non-heme proteins such as transferrins, ferritins, and flavin enzymes. It is necessary for the synthesis of hemoglobin, myoglobin, heme enzymes, and enzymes involved in oxidation and reduction processes [4,5]. Zinc is another metal present in the human body and is the second most abundant (about 2.5 g) trace element after Fe. Zn is an essential trace element that functions as a cofactor for certain enzymes involved in metabolism and cell growth; it is found in nearly 300 specific enzymes [6,7,8]. Chromium is found primarily in two forms: trivalent, which is biologically active and found in food, and hexavalent, a toxic form that results from industrial pollution. Cr (III) has been postulated to be involved in regulating carbohydrate, lipid, and protein metabolism by enhancing insulin efficacy [9,10]. Mg^2+^ is the second richest intracellular cation after K^+^ and is a cofactor in more than 325 enzyme systems in cells. Mg is used in many biological functions, where it functions as a cofactor in enzyme systems that regulate diverse biochemical reactions in the body, including protein synthesis, muscle and nerve functions, blood glucose control, and blood pressure regulation [1,11].

Micronutrients play a central role in metabolism and the maintenance of tissue functions [12]. In contrast to macronutrients (fat, carbohydrate, and protein), they cannot substitute for one another and cannot be synthesized within the body. Consequently, we depend on the delivery of all essential micronutrients via our diet [13].

Nowadays, plant products tend to be less nutrient-dense in comparison to plant products from previous decades. This is partly due to the progressive process of soil depletion as well as the introduction of highly productive plant varieties into agriculture [14]. These plants guarantee a high yield but are also characterized by low nutrient content. Therefore, the diet of an average human lacks vitamins and minerals such as iron, zinc, selenium, iodine, magnesium, calcium, and other nutrients. Macro- and micronutrient deficiencies affect a significant part of the world population. Growing concerns about the widespread impacts of micronutrient deficiencies have been magnified by the poor nutritional quality of crops that are less resistant to drought and other stresses, which are likely to be further exacerbated by continuing climate change [15,16]. It is estimated that 60–80%, 30%, 30%, and 15% of people suffer from deficiencies of Fe, I, Zn, and Se, respectively [17,18]. According to the WHO and FAO [19], we have to deal with so-called “hidden hunger” when food quality does not meet our nutrient requirements. The problem is so common that about two billion people are estimated to suffer from vitamin and mineral deficiencies.

An average human diet lacking in vitamins, minerals, or other nutrients poses a real challenge. The resolution is supposed to be found in plant product biofortification. Biofortification is understood as a process that increases the uptake and accumulation of mineral nutrients in agricultural products through the selection of agricultural practices, plant breeding, or/and product modification via genetic engineering [14,20,21,22]. The results should provide health benefits as well as pose a minimal risk for consumers [23]. Through biofortification, the nutrient content increases during crop growth rather than during crop processing. Commonly used practices are based on selecting fertilization and allowing the uptake of nutrients from the soil, or foliar fertilization [14].

Recently, a new approach for increasing micronutrient content in plants has been genetic engineering, which has been shown to be a feasible and cost-effective alternative to traditional fortification programs. In transgenic methods of fortification, new cultivars with desired traits can be developed by transferring new genes, overexpressing the genes already present, or blocking genes that provide inhibitor synthesis. These methods aim to transport and distribute micronutrients between tissues, thereby increasing their concentration in the edible parts of crops, and facilitate the efficiency and productivity of biochemical pathways involved in their synthesis. Transgenic biofortification requires time, effort, and investment to optimize the process. For that reason, this process requires making calculations as to whether it will pay off in long-term applications. There are also time-consuming and costly legal regulations regarding the commercial distribution of biofortified food crops, including difficulties in obtaining government approval, concerns and demands of anti-GMO activists for more tests before distribution, and low social acceptance. These main disadvantages of plant genetic engineering must be taken into account [24,25,26,27,28].

Plant products that are already rich in essential nutrients include crop sprouts. They are abundant in valuable components such as vitamins, amino acids, enzymes, dietary fibers, flavonoids, or antioxidants. Their high nutritional value classifies them as being highly desired in the human diet [29]. The biggest advantage of introducing sprouts to the human diet is the relatively short process of preparation as well as the possibility of biofortification.

The effectiveness of biofortification of edible seeds has been studied by several research teams. In the research by Park et al. [30] the effect of iron supplementation on alfalfa, broccoli and radish sprouts was analyzed. Iron concentration was supplemented by soaking seeds in Fe(III)-EDTA or Fe(III)-citrate solution at a concentration of 2.5, 5.0, or 10 mM. The soaking treatment significantly increased the iron concentration in 5-day-old alfalfa sprouts by up to 1.8 times the concentration observed in the controls. In broccoli and radish sprouts, the increase in Fe concentration was insignificant [30]. In other studies, the iron content increased by 1.1–15.6 times via soaking brown rice grains in an FeSO_4_ solution before germination compared to germinating the grains [31]. Se biofortification during sprouting could represent a valid strategy to improve Se concentration in tartary buckwheat sprouts [32] and brown rice [33]. In wheat, Se-enriched kernels can be obtained with 35 mg L^−1^ Na_2_SeO_3_ in a germination medium for 24 h at 25 °C [34].

The enrichment of plant sprouts with nutrients in hydroponic media does not require any special conditions [23,35]. However, there are still possibilities for the introduction of new materials for vessels for hydroponic cultivation [36] or new solutions for maintaining seed microbiological quality. Recently, researchers have conducted hydroponic cultivation of soybean, lettuce, garden cress, mung bean, broccoli, and onion sprouts or seedlings via biofortification [23,35,37,38,39]. These researchers noticed that enrichment with one element may affect the physiological and biochemical parameters of sprouts, including the assimilation of other micro- and macro-nutrients. Depending on the content of iodine in hydroponic media, the biofortified lettuce seedlings contained more potassium and less sodium and manganese than the control seedlings [35]. In the case of Zn, this element significantly influenced the Fe and Ca contents in soybean sprouts but did not affect the contents of Mn, Cu, and Mg [39].

Since common wheat (*Triticum aestivum* L.) is the most popular crop in the world and wheat sprouts show relatively high carbohydrate and amino acid values [29], we attempted to enhance the macro- and micro-nutrient densities of this food product. The aim of the present work was an evaluation of the ability to accumulate Fe, Zn, Mg, and Cr in germinated wheat seeds of two varieties, Tonacja and Arkadia, in hydroponic conditions. This work also aimed to assess the impact of seed surface sterilization via UV-C light on biofortification effectiveness and seed properties using a scanning electron microscope (SEM). The study performed using SEM provides information about the elemental composition of the samples in the microregion. Thus, it is possible to determine in which part of the plant the most biofortified element is deposited. The analysis of elemental composition in the microregion included a qualitative analysis of the test samples. The quantitative analysis of selected micronutrients in the plant tissues was performed using atomic absorption spectrometry (AAS) with flame atomization.

## 2. Results and Discussion

### 2.1. Sample Preparation and Macroscopic Analysis of Wheat Seeds 

Before sprouting, seeds are soaked and then maintained in a humid environment that is favorable for sprouting. Bacteria and associated biofilms grow well under conditions with enough moisture and nutrients. Thus, the sprouting stage has been categorized as a major source of bacterial contamination in sprouts because bacteria present in seeds can become internalized during the process of sprouting if they are not inactivated [40]. Since sprouts are consumed with minimal processing, it is extremely important to use effective decontamination methods that will ensure the elimination of pathogenic microbes and the safety of sprouts.

In our research, wheat seeds of the Tonacja and Arkadia varieties were sterilized with UV-C radiation in order to maintain microbiological purity. UV lamps (254 nm) were used in the tests, which are standard equipment in most laboratories. The minimum radiation dose (1.5 kJ m^−2^) was used in the experiments to inhibit the spread of yeasts/molds. This dose was experimentally optimized. Lower doses of UV-C did not inhibit infections caused by undesirable microorganisms during the experiment. The proposed UV treatment of seeds improves on current technology used for the production of hydroponic crops by taking into account modern requirements for energy savings and obtaining environmentally friendly products.

The influence of this non-chemical sterilization process on seeds was tested in an innovated manner via a scanning electron microscope and EXAKT thin-section cutting. The EXAKT system was developed in collaboration with Prof. Donath at the Institute for Pathology at the University of Hamburg in 1987, and since then, it has been widely used. The precise cutting leaves a perfect surface between different tissue structures. Burr-free, plane-parallel surfaces and thicknesses of down to 100 µm allow many slices to be cut in order to obtain as much structural information as possible from the original sample. In addition to organic specimen testing, the EXAKT system also finds application in the development of novel combinations of natural and synthetic materials used in industry or in recycling.

**Figure 1:**
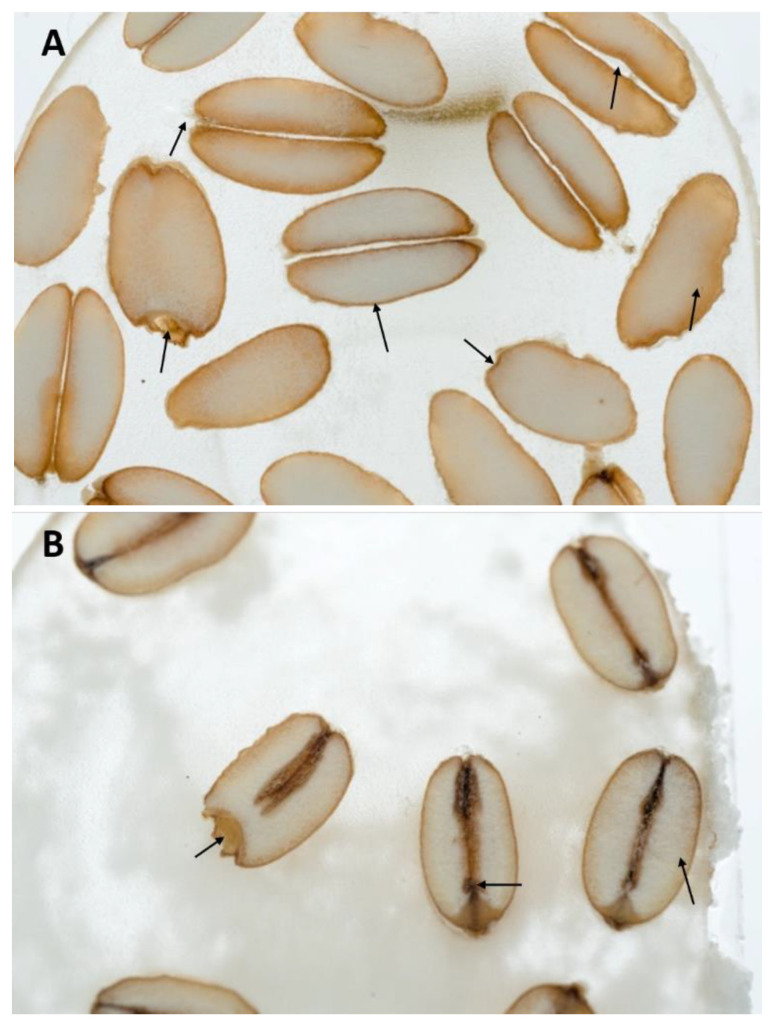
Macroscopic analysis of wheat seeds of the Tonacja variety treated (**A**) and not treated (**B**) with UV-C radiation as a factor for improving microbiological quality.

In this study, this method was adjusted for macroscopic analysis of plant seeds. It successfully allowed the observation of wheat grains of the Tonacja variety. The outer and internal structures were well captured. It allowed a visual separation of the outer layer, including the pericarp, brush, crease, starchy endosperm with color gradient, pigment stand, and embryo with scutellum, radicle, and coleoptile (Figure 1A,B).

Between the two tested specimens, there were no significant changes in morphology. This indicated that wheat seed sterilization for 30 min using UV-C radiation was not a too harsh treatment (Figure 1A).

**Figure 2:**
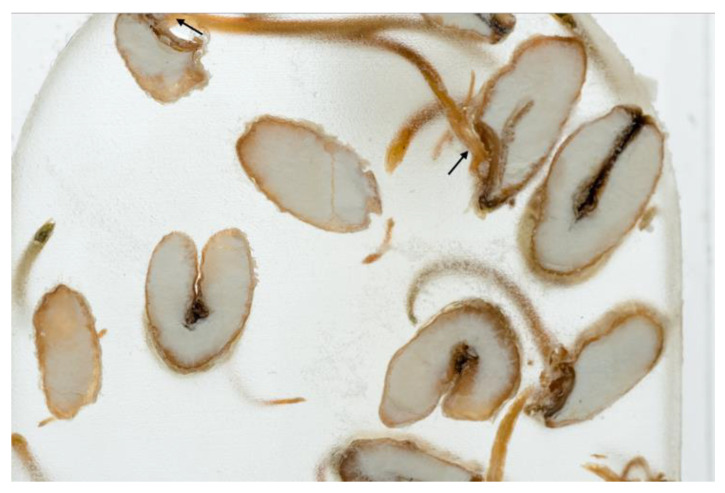
Morphological changes in wheat seeds caused by germination mechanisms.

The presented approach appeared to be suitable for following the morphological changes caused by the seed germination process and sprout formation as well. Figure 2 presents the hypocotyl development.

### 2.2. Seed Germination Energy

It has been proven that wheat germination efficiency is affected by the presence of microbiological and mineral components in hydroponic media [41]. In this study, the type of ion enrichment did not affect the seed germination energy (Table 1). An exception is Cr biofortification, which leads to a reduction in germination energy (79–88%) in comparison to the control samples (94–100%). Cr is responsible for reactive oxygen species production. It results in lipid, protein, and nucleic acid degradation in plants. That may lead to seed germination, photosynthesis, or water balance dysfunction [42,43,44].

The more prominent differences in seed germination energy (Table 1) are mainly associated with the plant material treatment used. Previous experiments have shown that UV-C radiation stimulates the germination of edible seeds, including wheat seeds [45]. In this experiment, UV-C radiation reduced the seed germination energy and noticeably suppressed yeast/mold spread (data not shown). This result leads to the conclusion that microflora may promote germination of wheat seeds in the early stages of infection (within four days). The results reported by other authors reveal diverse relations between wheat seed germination energy and the type of microflora in the environment, including a non-significant difference in germination between healthy and infected seeds due to infection by *Alternaria alternate* [46], negative effects induced by fungal seed-borne pathogens (*Alternaria tenuis, Aspergillus niger, Fusarium moniliforme, Curvuluria lunata,* and *Stemphylium herhurum*) [47], and positive effect caused by bacteria [41].

It is difficult to clearly conclude whether some changes may be induced primarily by the lack/presence of microflora or by UV-C radiation itself. It should be noted that long-term exposure to UV light shortens the germination process and subsequently deforms the seedlings [48]. Permanent exposure may interrupt germination altogether. After 30 min of seed treatment with UV-C (λ = 254), we noticed any physiologically incorrect plant growth (Table 2 and Table 3).

In this experiment, no significant differences in germination energy between the UV-exposed seeds of the wheat varieties Tonacja and Arcadia were detected. Both show exceptionally efficient seed germination energy.

### 2.3. The Influence of Seed UV-C Sterilization on Biofortification Efficiency

The microbiological quality of seeds during the germination process must be maintained. This is especially important when the plant material is susceptible to fungal diseases, as in the case of common wheat [49]. We used a non-chemical treatment for seed surface sterilization by UV-C radiation and evaluated its impact on biofortification efficiency.

To confirm the effectiveness of biofortification and to demonstrate that the quantified content of trace elements does not come from grain surface contamination but from an effective enrichment process, a microscopic analysis was performed, and selected samples with the best morphological characteristics were tested. The samples for the SEM examination were biofortified wheat varieties (100 µg g^−1^) and a control sample. The analysis with a scanning electron microscope allowed us to obtain high-resolution images and maps of the distribution of elements. Examples of the SEM analysis results obtained for the control sample and the Mg-biofortified sample are presented in Table 4.

**Figure 3:**
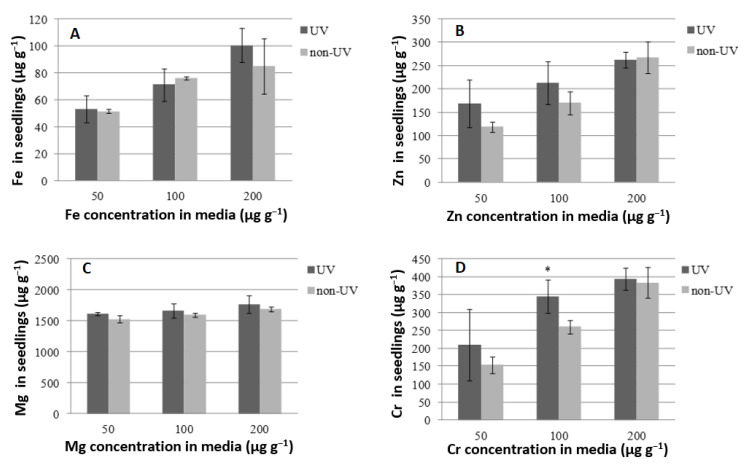
The influence of seed UV-C sterilization on micronutrient concentration in the wheat sprouts of the Tonacja variety. * refers to a result that is significantly different at the level of *p* < 0.05 according to Tukey’s test. Data are presented as mean ± SD.

The quantitative analysis of selected micronutrients in plant tissues was performed using atomic absorption spectrometry with flame atomization. In this experiment, we observed slightly better assimilation of Cr and Zn from the hydroponic media into the sprout tissues of the sample treated with UV-C radiation (Figure 3). Only medium supplementation with chromium (100 µg g^−1^) significantly improved the element concentration in sprouts. Without medium enrichment, Cr is detectable only in wheat seeds infected by microorganisms [50]. Based on this study, a lack of seed surface sterilization (the presence of microflora) results in insignificantly lower Cr assimilation in sprout tissues. UV-C sterilization had no influence on Fe and Mg concentrations in the tested sprouts. The study by Blicharska et al. [23] reported that there is a slight correlation between the occurrence of microbiological contamination of sprouts and the presence of a particular type of ion in the hydroponic media or tested plant species.

This study is the first to feature UV-C irradiation as a factor for seed surface sterilization and to correlate it with the level of nutrient uptake by sprouts or seedlings. Thus far, seed treatment with low doses of UV has proven to induce various morphological and physiological changes in the later stages of plant growth. This method improves plant productivity and yield quality [51,52,53]. It activates mechanisms of biotic and abiotic stress adaptation in plants by, e.g., stimulating the synthesis of antioxidants in leaves [52,54,55]. It may affect photosynthesis and the content of chlorophyll in leaves [51,56]. Pre-sowing UV-C irradiation of wheat seeds may result in seed coat thicknesses that relate to better resistance against pathogens [51]. Most of the studies have focused on post-sowing UV-A/B radiation [57]. It is considered a stress factor connected with stratospheric ozone layer depletion. Under natural conditions, ozone loss may affect plant growth, including the uptake of nutrients by wheat seedlings [58].

### 2.4. Wheat Biofortification with Nutrients

As shown in Table 5, the ion type and concentration in the media affect the final concentrations of Fe, Zn, Mg, and Cr in the wheat sprouts. We correlated this result with a visual assessment of the plant materials (Table 2) in order to select the optimum conditions for wheat biofortification with macro- and micro-nutrients. Even if vegetables or cereals are able to assimilate a great amount of particular nutrients, the final, maximum concentration may be toxic to seedlings/sprouts.

**Figure 4:**
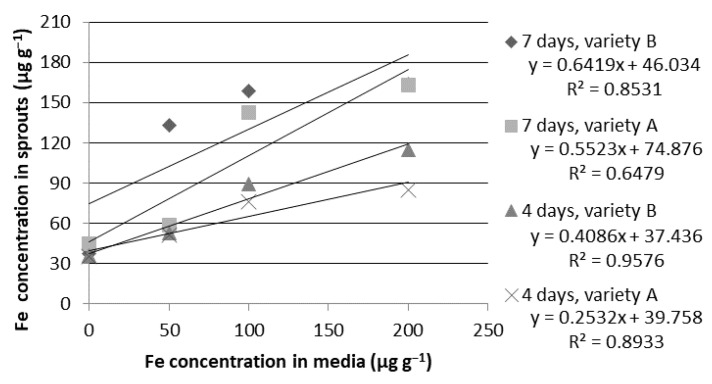
Relationship between iron (Fe) concentrations in the growth media and in the dry weight of the wheat sprouts of the Tonacja (A) and Arkadia (B) varieties (µg g^−1^).

**Iron.** In comparison to other nutrients, the correlation between the ion concentration in the media and the accumulation of this element in the plant material is the weakest. The linear relationship becomes even weaker over time (Figure 4). For the Tonacja variety, the coefficient of determination decreases from R^2^ = 0.8933 to R^2^ = 0.6479, and for the Arkadia variety, it decreases from R^2^ = 0.9576 to R^2^ = 0.8531. However, this result is still promising. The morphological assessment revealed that the most healthy-looking sprouts were grown in the solution with a concentration of 100 µg g^−1^.

The concentration of 200 µg g^−1^ significantly reduced wheat development, which expressed shorter hypocotyl and physical sprout deformation (Table 2). After four days of hydroponic cultivation in the 100 µg g^−1^ solution, there was an increase in 117% and 150% in iron accumulation for the Tonacja and Arkadia varieties, respectively. The 7-day cultivation resulted in 218% and 322% better nutrient accumulation in comparison to the control condition, respectively. Further supplementation (200 µg g^−1^) significantly increased biofortification effectiveness only for the Arkadia variety on the fourth (114.96 µg g^−1^) and seventh days (163.47 µg g^−1^) of cultivation in comparison to the lower dose (100 µg g^−1^). The primary Fe concentration in these wheat grains was at a similar level (35–44 µg g^−1^) to that in most wheat crops [47].

Several methods to correct Fe deficiency, such as broadcast application, foliar spray, and seed priming (soaking seeds in a nutrient-rich solution before sowing), have been used [59].

A previous study showed that foliar application of Fe at each of the different growth stages of wheat significantly increased grain yield and the concentration of Fe in grains. Foliar feeding involves applying a fertilizer spray in a liquid state over leaves. A single micronutrient solution or a mixture of such solutions in combination with the target concentration of salt is applied as a spray on leaves, where it is absorbed via stomata and epidermis. In particular cases, foliar feeding is shown to be more efficient when compared to soil applications for the effective uptake of nutrients. In one study, foliar sprays of Fe (FeSO_4_·2H_2_O) were applied at different growth stages of wheat, starting from the maximum tillering, flower initiation, milk, and dough stages [60]. A similar strategy has been used with other edible grains [61].

However, in the case of biofortification of sprouts (taking into account the specificity of the products and the method of their cultivation), the strategy of Fe supplementation by soaking seeds in appropriate solutions seems to be more appropriate. Seed priming involves the controlled hydration of seeds that permits them to perform their pre-germination metabolic events without radical emergence. This treatment activates enzymatic and metabolic processes, which may enhance the plant’s capacity for nutrient uptake and their subsequent translocation [59].

**Figure 5:**
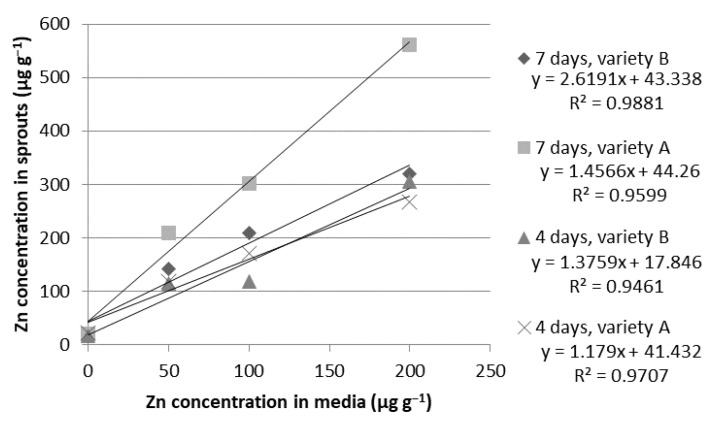
Relationship between zinc (Zn) concentrations in the growth media and in the dry weight of the wheat sprouts of the Tonacja (A) and Arkadia (B) varieties (µg g^−1^).

**Zinc.** The Zn concentration in wheat sprouts was very strongly correlated with the ion concentration in the media. Regardless of the cultivation time or plant variety, the determination coefficient was higher than R^2^ = 0.940 (Figure 5). This nutrient assimilation was also the most effective. A similar effect of biofortification of various vegetable sprouts was previously noticed by Blicharska et al. [23].

Even the smallest dose of 50 µg g^−1^ caused an approximately 5–7 fold increase in the Zn sprout content during the first step of the experiment. The final Zn concentration after seven days was approximately 8–11 times higher in comparison to the control (0 µg g^−1^). The enrichment of the hydroponic media with a solution of 100 µg g^−1^ initially induced an 8–11 fold increase in Zn concentration in the plant tissues; afterward, it reached a 12–15 fold higher level in comparison to the control condition. The maximum wheat biofortification was observed with a solution of 200 µg g^−1^. At the end of hydroponic cultivation (seven days), the sprouts of the Arkadia (320.06 µg g^−1^) and Tonacja (560.53 µg g^−1^) varieties were 19 and 29 times richer in zinc compared to the sprouts without supplementation (17–20 µg g^−1^) (Table 5). The morphological response of the sprouts was strongly correlated with the type of plant. The sprouts of the Tonacja variety were characterized by very stable growth compared to the Arkadia variety. These sprouts were longer and better developed. We noticed that the sprouts, especially of the Tonacja variety, were in visibly better condition when grown in the hydroponic media with a 100 µg g^−1^ concentration. The solution of 200 µg g^−1^ was associated with uneven growth of sprouts and inhibition of root elongation (Table 2). These results indicate that the Tonacja variety is more resistant to the toxic effects of this element at high concentrations.

There is evidence in the literature demonstrating that foliar-applied Zn can be absorbed by the leaf epidermis and then remobilized and transferred into rice grains through the phloem [62]. In recent years, several studies have been conducted to adjust the time of foliar Zn application in cereal crops [63,64,65]. It is now well established that foliar Zn application after the flowering stage (e.g., at the early milk and dough stages) more distinctly increases the Zn concentration in grains [64]. However, the process of enriching of crop sprouts under hydroponic conditions is the easiest and cheapest to conduct and control.

**Figure 6:**
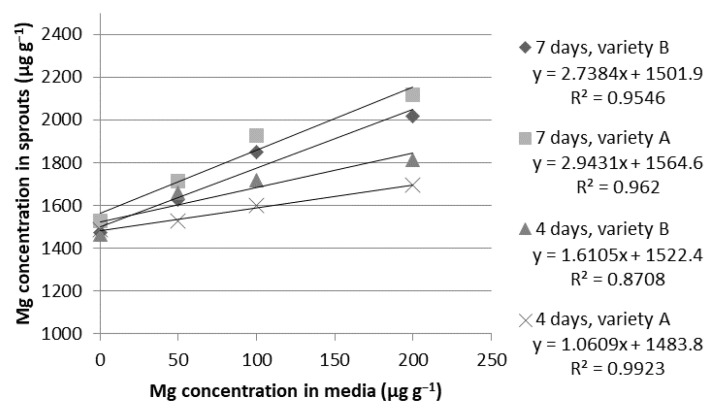
Relationship between magnesium (Mg) concentrations in the growth media and in the dry weight of the wheat sprouts of the Tonacja (A) and Arkadia (B) varieties (µg g^−1^).

**Magnesium.** There was a strong correlation between the Mg concentrations in the media and in the wheat sprouts (Figure 6). It was most pronounced for the seeds of the Tonacja variety cultivated by the fourth day (R^2^ = 0.9923) and weakest for the seeds of the Arkadia variety tested at the same time (R^2^ = 0.8708). The enrichment of the hydroponic media with the ion solution at a concentration of 50 µg g^−1^ did not significantly affect the biofortification level, regardless of the sampling time. Using solutions of 100 and 200 µg g^−1^ in the first stage of the experiment (four days) resulted in a similar level of Mg concentration in the sprouts of the Tonacja (1599–1693 µg g^−1^) and Arkadia varieties (1717–1814 µg g^−1^). After seven days, we noticed significant improvement in Mg assimilation with increasing ion concentration in the media from 1928 µg g^−1^ to 2118 µg g^−1^ for the Tonacja variety and from 1851 µg g^−1^ to 2015 µg g^−1^ for the Arkadia variety. Overall, the maximum plant product biofortification with magnesium did not exceed 40% in intensity when compared to the control sample (Table 5). The relatively low percentage of magnesium absorption is reflected in the morphological condition of the sprouts (Table 2). Both the Tonacja and Arkadia varieties showed significantly better sprout growth with an increased concentration of Mg in the hydroponic solution. At concentrations of 100 and 200 µg g^−1^, we observed long sprouts with a well-developed root system. However, it could be observed that the roots formed in the medium supplemented with 200 µg g^−1^ of magnesium are slightly shorter in comparison to the roots grown in a solution containing 100 µg g^−1^ of magnesium.

**Figure 7:**
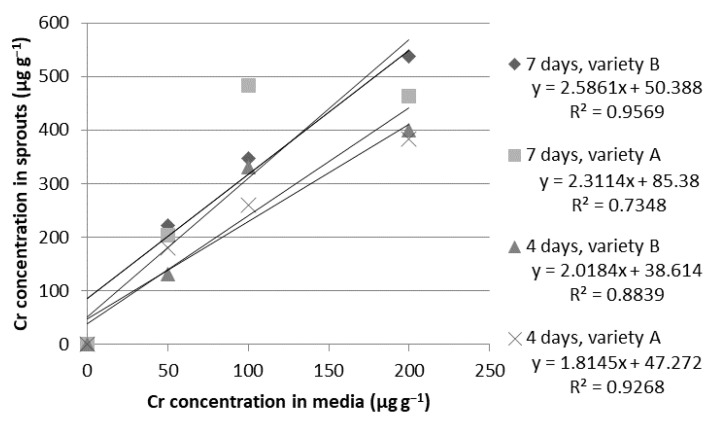
Relationship between chromium (Cr) concentrations in the growth media and in the dry weight of the wheat sprouts of the Tonacja (A) and Arkadia (B) varieties (µg g^−1^).

**Chromium.** According to Singh et al. [50] and our results, Cr does not occur naturally in wheat grains. This element is highly toxic to wheat plants. It interferes with various metabolic processes and leads to the inhibition of plant growth and development [43]. Our results show that wheat assimilates chromium very effectively. Chromium concentrations after four days of cultivation reached 179.79, 260.58, and 383.80 µg g^−1^ for the Tonacja variety and 130.42, 331.35, and 399.12 for the Arkadia variety when grown in the ion solutions of 50, 100, and 200 µg g^−1^, respectively. The maximum wheat biofortification with Cr was achieved after seven days of cultivation. The sprouts of the Tonacja variety reached the highest micronutrient concentration (483.30–463.60 µg g^−1^) in the presence of 100 and 200 µg g^−1^ of ion solution. In contrast, the Arkadia variety assimilated 538.19 µg g^−1^ of Cr when cultivated in the hydroponic medium supplemented with 200 µg g^−1^ of ion solution (Table 5). In this case, we also noted a strong linear correlation between the ion concentration in the media and the accumulation of Cr in the plant material (R^2^ = 0.9569) (Figure 7). The morphological assessment revealed that the Arkadia variety of wheat has a greater tolerance for the toxic properties of this element. In the presence of Cr at concentrations of 50 and 100 µg g^−1^, the sprouts of the Arkadia variety were more healthy-looking. They were characterized by better developed hypocotyls and root systems. Regardless of the plant type, the best-developed sprouts were grown in the solution with 50 µg g^−1^ of chromium. In contrast, the concentration of 200 µg g^−1^ was clearly toxic to the wheat sprouts. The sprouts were significantly shorter. Root growth was also inhibited. For many seeds, a complete inhibition of germination was noticed (Table 2).

Since wheat is the most popular crop in the world, there is a high interest in increasing its nutritional value [66]. The grains of cereal crops like wheat contain various nutrients, such as phytic acid and tannins. Phytic acid forms a complex with mineral elements and reduces Fe and Zn bioavailability. This leads to a deficiency of these nutrients in the human body via their uptake through diet [67,68]. Overall, the percent bioavailability of Fe and Zn in seeds and grains of full-grown cereal crops is as low as 5% and 25%, respectively [69]. In this experiment, we obtained significantly better results when testing their bioavailability in wheat sprouts. The maximum assimilation of Fe and Zn reached 322% and 1442%, respectively, when the optimal ion concentration was applied (100 µg g^−1^). About 90% of Mg from hydroponic media is stored in leaves, not grains [70]. This confirms that sprouts are a fast, easily available, and relatively safe source of nutrient supplementation [38].

## 3. Materials and Methods

### 3.1. Ion Solutions and AAS Standards

In order to prepare the solutions of Zn, Cr, Fe, and Mg, the following chemicals were used: zinc nitrate hexahydrate (Zn(NO_3_)_2_·6H_2_O), chromium (III) sulfate hydrate (Cr_2_(SO_4_)_3_·18H_2_O), iron (III) chloride hexahydrate (FeCl_3_·6H_2_O), and magnesium nitrate hexahydrate (Mg(NO_3_)_2_·6H_2_O). For every individual metal, concentrations of 50, 100, and 200 µg g^−1^ were prepared in solutions with a volume of 200 mL. The prepared solutions were sterilized under a UV-C lamp for 30 min before their use.

The AAS ion standards for zinc, chromium, iron, and magnesium at a concentration of 1000 µg L^−1^ (Merck, Darmstadt, Germany) were acquired. For the quantitative analysis, solutions at varying concentrations, depending on the primary metal concentration in the plant material, were prepared via the dilution of appropriate standards. The following dilutions (µg g^−1^) of Fe^3+^ (0.3, 2.5, 5.0, 7.5, and 10.0), Zn^2+^ (0.1, 0.3, 0.5, 0.8, and 1.0), Mg^2+^ (0.5, 1.2, 1.8, 2.4, and 3.0), and Cr^3+^ (0.1, 0.2, 0.3, 0.4, and 0.5) were prepared.

### 3.2. Plant Material

The plant material selection was made carefully. For the purpose of this experiment, two varieties of common wheat, *Triticum aestivum* var. Tonacja (variety A) and *Triticum aestivum* var. Arkadia (variety B), with short germination times and high nutrient values, were chosen. The research material was obtained from the research farm in Czesławice (Poland). Both varieties were reported to have great popularity and wide application. The seeds were pure and free of preservatives.

### 3.3. UV-C Exposure

Sprouts are desired to be incorporated into human diet routines, and as with other edible materials, they need to meet some quality standards. A portion of the wheat seeds underwent a non-chemical sterilization process with a UV-C lamp (λ = 254 nm) for 30 min in order to maintain microbiological quality and safety. The UV lamp was TUV 15W/G15 T8 type (Philips, Eindhoven, The Netherlands), and 15 W power was used as the irradiation source. The distance between the lamp and the samples was 25 cm. The intensity of energy irradiation reached 0.85 W m^−2^. The measurements of UV-C radiation dose were conducted using a UVC-2GRM radiometer (Sonopan, Białystok, Poland).

### 3.4. Sample Preparation and Analysis of Wheat Seeds via Scanning Electron Microscopy (SEM)

In order to trace the features of wheat seeds affected by UV-C light exposure, a new approach was introduced. In this study, the well-known method of scanning electron microscopy (SEM), together with sample preparation, was altered for this specific plant material. The wheat seeds were submerged in plastic resin and subsequently cut into thin layers. In this process, the optimization of light polymerization was challenging because many factors play a role in the preservation of organic material structures when they are embedded in resin. To avoid blistering and cracking, the curing time, degree of curing, and temperature development must be controlled and adapted to the type and origin of the material.

In this study, the polymerization of the wheat seeds of the Tonacja variety (treated and not treated with UV-C radiation) in the Technovit^®^ 2000 LC resin provided by Heraeus Kulze (Wehrheim, Germany) was carried out using Light Polymerizer Exakt 520 (Exakt, Hamburg-Norderstedt, Germany). The following conditions were incorporated into the polymerization process: an exposure time of 45 min and the use of blue light (the bulb type was 9W/71).

Furthermore, the high-precision EXAKT thin-section cutting and grinding system was introduced. It was used for the preparation of the sections, with a thickness down to 10 µm to retain the native morphology of the samples. The method of thin-section cutting is especially important in the study of the surface of biomaterials since the tested area can be prepared while preserving the overall structure, and light microscopic techniques can be used directly, e.g., SEM. The use of the saw-like EXAKT 312 allows the quick and precise preparation of the native condition of a sample and creates a precondition for further diagnostics.

In the present experiment, the resin blocks were cut using a Diamond Bend Saw EXAKT 312 (Exakt, Hamburg-Norderstedt, Germany) with a diamond band. The cutting time was 60 s, and the depth of intersection was 2 mm. The type of diamond band used to cut the samples was Exakt 0.3/D151.

The samples were prepared with the courtesy of the company AMP Medical, which is the exclusive distributor of Exakt machines in Poland.

The microarea studies were carried out using a Hitachi SU6600 (Hitachi, Tokyo, Japan) scanning electron microscope. The samples were tested without sputtering under low vacuum conditions (5–10 Pa). As part of the analysis of the samples, microphotographic documentation was made using the backscattered electron (BSE) technique; then, using an EDS adapter with a silicon detector provided by Thermo (Waltham, MA, USA), the chemical composition of the tested samples was analyzed. The analysis was carried out at a voltage of 25 kV, and the time of data collection was 90 s. The working distance was about 10 mm, and the magnification was about 100–500 times.

### 3.5. Hydroponic Cultivation

The experiment was performed under hydroponic conditions in a thermostatic chamber. Plates that were 80 mm in diameter were coated with wet filter paper. The plates with seeds were incubated at room temperature (21–23 °C), with a relative humidity of 60–70% and natural light exposure (day/night). The appropriate humidity level and medium concentration were maintained by systematic refilling. An equal number of seeds (100) per plate was placed directly on the surface of filter papers soaked with ion solutions at four different concentrations: 0 (control), 50, 100, and 200 µg g^−1^ for each nutrient. The incubation was performed between 4 and 7 days. Additional protocols, including the cultivation of unsterilized wheat seeds of variety A for 4 days in the four hydroponic media with the four elements at a concentration of 100 µg g^−1^, were performed. The experiments were carried out in three independent replications for each treatment. During the experiment, sprout development as well as microbiological infection spread were precisely monitored.

### 3.6. Seed Germination Energy

The seed germination energy was determined on the basis of the International Seed Testing Association protocol (ISTA, Essen, Germany) [71]. For this purpose, we used the seeds of varieties A and B that were treated with UV-C radiation (UV-treated) and the seeds of variety A that were free from any surface sterilization (non-UV-treated). The germinated seeds were counted at 24, 48, and 96 h after sowing in the hydroponic media with 100 µg g^−1^ of ion solutions (Fe, Zn, Mg, and Cr) and control (0 µg g^−1^). The fraction of germinated seeds (number of sprouts) after 4 days of germination was defined as the germination energy *G*. The threshold was at least 2 mm for the long radicle [71]. The germination energy was expressed as a fraction of the germinated seeds *G* after a defined time *t* and calculated using the following equation:

(1) $$G=\frac{n}{n_{T}}\times 100\%$$

where *n* is the number of seeds germinated at time *t*, and *n_T_* is the total number of sown seeds.

### 3.7. Collection and Mineralization of Sprout Samples

After 4 and 7 days of incubation, the sprouts were washed several times with deionized water. This allowed the removal of metal ions absorbed on their surface. The samples were dried at 60–65 °C to an equal weight. The next step was sample mineralization with the use of a Mars 5 microwave-accelerated reaction system with XP1500 vessels (vol. 50 milliliters, max. 55 bar) obtained from CEM (Kamp-Lintfort, Germany). A total of 0.5 g of dried material was placed into an extraction vessel. The sample digestion was performed in the presence of 3 mL of 69% nitric acid and 7 mL of deionized water. The mineralization procedure included a temperature–time ramp for 10 min with a final temperature of 180 °C that was held for 20 min and a cooling step lasting 15 min. The plant material was then suspended in deionized water to a volume of 25 mL.

### 3.8. The AAS Conditions

The accumulation of micronutrients in the plant material was tested using high-resolution continuum source flame atomic absorption spectrometry (Analytik Jena AG, Germany) in the presence of acetylene as the gas fuel and air as an oxidant. The optimal wavelength (nm) for each element was as follows: Fe at 248.3, Zn at 231.9, Mg at 285.2, and Cr at 357.9. The reference solution was prepared with 0.5% nitric acid and 0.5 mL of 19% KCl in a volume of 100 mL. The signals reported as the peak areas were analyzed using the Aspect CS 2.0.0 software (Analytik Jena AG, Jena, Germany) and calculated as the ion content (µg) per sprout of dry matter (g). The calculation included the material volume after mineralization and the dilution used for a particular sample.

### 3.9. Statistical Analysis

One-way analysis of variance (ANOVA) was performed using the Statistica 10.0 software, with a level of significance of *p* < 0.05. Homogeneous groups were determined using Tukey’s test.

## 4. Conclusions

The obtained results confirmed that both tested common wheat (*Triticum aestivum* L.) varieties (Arkadia and Tonacja) have a high ability to accumulate Fe, Zn, Mg, and Cr during the germination process. The highest feasibility was indicated for Zn biofortification (R^2^ > 0.940). For other elements, a very strong correlation between the ion concentration in the media and their bioassimilation in the plant tissues was also noticed (R^2^ > 0.90). The ion concentration of 100 µg g^−1^ was assumed to be the most appropriate for effective bioaccumulation of ions in the wheat sprouts. After seven days of hydroponic cultivation in the 100 µg g^−1^ solution, there was an increase in iron accumulation of 218% and 322% in the plant materials of the Tonacja and Arkadia varieties, respectively. The final Zn concentration after seven days was approximately 8–11 times higher in comparison to the control. The ion dose of 200 µg g^−1^ showed high potential in producing nutrient-rich sprouts; however, it reduced sprout biomass growth.

The applied UV-C seed sterilization was proven to be an effective factor in preventing infections during seed germination. UV-C irradiation reduced the seed germination energy by 5–9%, depending on the type of ion present in the hydroponic media. However, it did not have an adverse effect on the morphological features of wheat grains or future sprouts, as well as on nutrient assimilation, and it could even significantly improve the biofortification of Cr.

In summary, this study showed that the conditions applied could be an effective tool for obtaining fortified food in a cheap and quick way.

## Figures and Tables

**Table 1:** Wheat seed germination energy in the hydroponic media with the presence of Fe, Zn, Cr, and Mg at a concentration of 100 µg g^−1^: (A) Tonacja and (B) Arcadia. Data are presented as mean ± SD.

Ion Concentration in Media (100 µg g^−1^)	Seed Germination Energy (%)		
	Variety A (Non-UV)	Variety A (UV)	Variety B (UV)
0 (control)	100.0 ± 0.0	94.3 ± 0.6	95.3 ± 1.5
Fe^3+^	98.3 ± 0.6	91.0 ± 0.0	91.0 ± 0.0
Zn^2+^	97.0 ± 0.0	89.7 ± 1.2	92.0 ± 0.0
Mg^2+^	97.7 ± 1.5	89.3 ± 0.6	90.3 ± 0.6
Cr^3+^	88.0 ± 1.7	80.7 ± 1.5	79.3 ± 1.5

**Table 2:** Morphological assessment of wheat sprouts in the hydroponic media with the presence of Fe, Zn, Cr, and Mg at four concentrations (0, 50, 100, and 200 µg g^−1^): (A) Tonacja and (B) Arcadia, with −/+ indicating physiologically correct growth, - indicating weaker growth or slightly deformation, -- indicating substantial deformation or inhibition of seed germination, + indicating faster growth, and ++ indicating substantial longer sprouts.

Ion Concentration in Media (µg g^−1^)		Morphological Features of Sprout		
		Variety A (Non-UV)	Variety A (UV)	Variety B (UV)
Fe	0 (control)	−/+	−/+	−/+
	50	−/+	−/+	−/+
	100	+	+	+
	200	-	-	-
Zn	0 (control)	−/+	−/+	−/+
	50	−/+	+	−/+
	100	+	++	+
	200	−/+	−/+	-
Mg	0 (control)	−/+	−/+	−/+
	50	−/+	−/+	−/+
	100	+	+	+
	200	+	+	+
Cr	0 (control)	−/+	−/+	−/+
	50	−/+	−/+	+
	100	−/+	−/+	+
	200	--	--	--

**Table 3:** Morphological appearance of the *Triticum aestivum* variety Tonacja treated with UV-C and grown in hydroponics supplemented with different concentrations of bioelements.

Elements	Concentrations of Elements (µg g^−1^)		
	50	100	200
Iron (Fe)	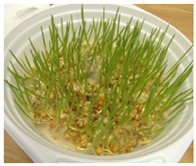	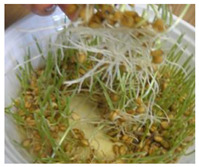	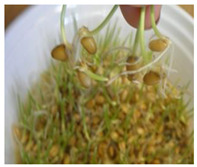
Zinc (Zn)	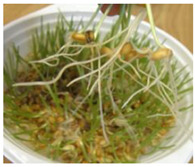	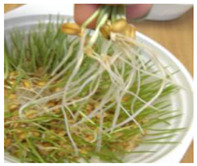	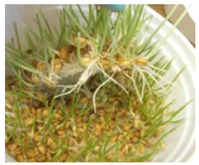
Magnesium (Mg)	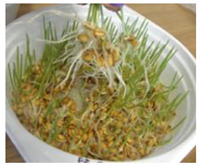	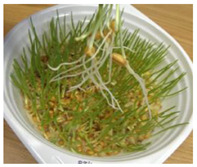	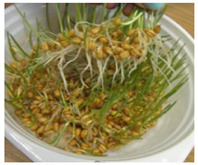
Chromium (Cr)	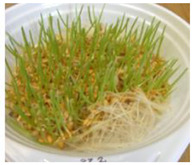	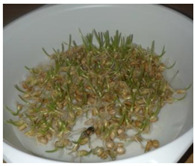	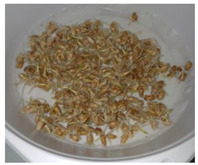
Control ^1^	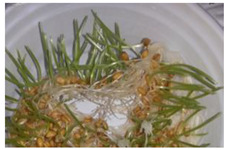		

^1^ Control—medium without bioelement supplementation.

**Table 4:** SEM analysis results obtained for the control sample and the sample of the *Triticum aestivum* variety Tonacja biofortified with Mg.

Mg Concentration in Media	BSE Micrograph with Graphic Arrangement of Measurement Points Made by SEM	Element Distribution Maps
0 µg g^−1^ (Control)	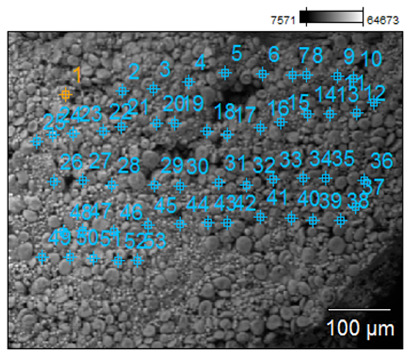	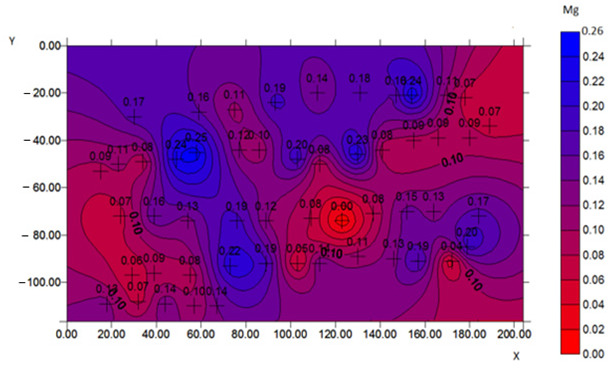
100 µg g^−1^	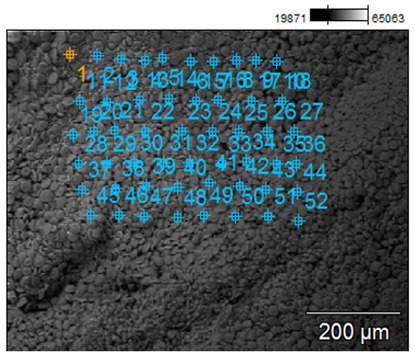	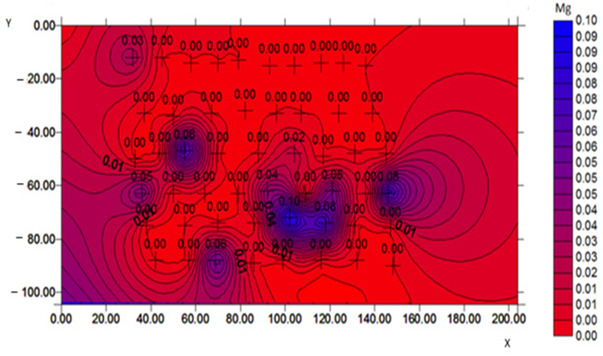

**Table 5:** Nutrient concentrations in the dry weight of the wheat sprouts of Tonacja (A) and Arkadia (B) varieties (µg g^−1^) with the presence of Fe, Zn, Cr, and Mg in the hydroponic media at four concentrations (0, 50, 100, and 200 µg g^−1^). Different letters within each column indicate significant differences at *p* < 0.05 based on Tukey’s test. Data are presented as mean ± SD.

Ion Concentration in Media (µg g^−1^)		Ion Concentration in Sprouts (µg g^−1^)			
		4 Days		7 Days	
		Variety A	Variety B	Variety A	Variety B
Fe	0 (control)	34.96 ± 3.96 c	35.65 ± 0.85 d	44.77 ± 2.30 c	37.55 ± 2.43 c
	50	51.61 ± 1.36 bc	53.03 ±2.83 c	58.36 ± 3.02 c	133.40 ± 8.37 b
	100	75.89 ± 1.15 ab	89.13 ± 9.79 b	142.41 ± 3.91 b	158.40 ± 9.26 a
	200	85.20 ± 20.5 a	114.96 ± 3.44 a	163.27 ± 9.55 a	163.47 ± 7.25 a
Zn	0 (control)	22.30 ± 2.09 c	16.11 ± 0.92 c	19.54 ± 0.64 d	16.72 ± 0.17 d
	50	118.79 ±10.48 b	114.29 ± 1.96 b	208.64 ± 45.71 c	141.14 ±13.10 c
	100	169.99 ±24.30 b	117.44 ± 12.76 b	301.32 ± 11.64 b	208.92 ± 24.52 b
	200	267.31 ±34.61 a	305.12 ± 64.95 a	560.53 ± 16.75 a	320.06 ± 34.15 a
Mg	0 (control)	1486.32 ± 7.92 c	1464.48 ± 70.78 c	1527.41 ± 60.30 d	1473.89 ± 31.30 c
	50	1527.18 ±55.21 bc	1657.53 ± 72.16 b	1715.02 ± 13.35 c	1625.96 ±57.53 c
	100	1599.22 ±33.41 ab	1717.31 ± 42.40 ab	1928.44 ± 34.53 b	1851.03 ± 92.55 b
	200	1693.68 ± 39.14 a	1813.90 ± 31.11 a	2117.65 ± 111.59 a	2015.14 ± 32.52 a
Cr	0 (control)	0.00 ± 0.00 d	0.00 ± 0.00 c	0.00 ± 0.00 c	0.00 ± 0.00 d
	50	179.79 ± 22.79 c	130.42 ± 13.88 b	203.60 ± 7.26 b	221.64 ± 33.74 c
	100	260.58 ± 19.13 b	331.35 ± 10.82 a	483.30 ± 28.46 a	346.85 ± 63.34 b
	200	383.80 ± 43.85 a	399.12 ± 79.01 a	463.60 ± 31.99 a	538.19 ± 10.71 a

## Data Availability

Not applicable.

## References

[1] Sigel A., Sigel H., Sigel R.K.O. (2006). Neurodegenerative Diseases and Metal Ions.

[2] Lee T.G., Park J.-W., Shon H.K., Moon D.W., Choi W.W., Li K., Chung J.H. (2008). Biochemical imaging of tissues by SIMS for biomedical applications. Appl. Surf. Sci..

[3] Al-Fartusie F., Mohssan S. (2017). Essential Trace Elements and Their Vital Roles in Human Body. Indian J. Adv. Chem. Sci..

[4] McDowell L.R. (2003). Minerals in Animal and Human Nutrition.

[5] Hurrell R.F. (1997). Bioavailability of iron. Eur. J. Clin. Nutr..

[6] Osredkar J. (2011). Copper and Zinc, Biological Role and Significance of Copper/Zinc Imbalance. J. Clin. Toxicol. S.

[7] Prasad A.S. (2003). Zinc deficiency: Has been known of for 40 years but ignored by global health organisations. BMJ.

[8] Plum L.M., Rink L., Haase H. (2010). The essential toxin: Impact of zinc on human health. Int. J. Environ. Res. Public Health.

[9] Mertz W. (1993). Chromium in human nutrition: A review. J. Nutr..

[10] Vincent J.B., Lukaski H.C. (2018). Chromium. Adv. Nutr..

[11] Kostov K., Halacheva L. (2018). Role of Magnesium Deficiency in Promoting Atherosclerosis, Endothelial Dysfunction, and Arterial Stiffening as Risk Factors for Hypertension. Int. J. Mol. Sci..

[12] Shenkin A. (2006). Micronutrients in health and disease. Postgrad. Med. J..

[13] Biesalski H.K., Tinz J. (2018). Micronutrients in the life cycle: Requirements and sufficient supply. NFS J..

[14] Yin X., Yuan L., Lin Z., Yin X., Yuan L. (2012). Phytoremediation and Biofortification. Two Sides of One Coin. Phytoremediation and Biofortification.

[15] Huey S.L., Krisher J.T., Bhargava A., Friesen V.M., Konieczynski E.M., Mbuya M.N.N., Mehta N.H., Monterrosa E., Nyangaresi A.M., Mehta S. (2022). Review of the Impact Pathways of Biofortified Foods and Food Products. Nutrients.

[16] Beach R.H., Sulser T.B., Crimmins A., Cenacchi N., Cole J., Fukagawa N.K., Mason-D’Croz D., Myers S., Sarofim M.C., Smith M. (2019). Combining the effects of increased atmospheric carbon dioxide on protein, iron, and zinc availability and projected climate change on global diets: A modelling study. Lancet Planet Health.

[17] Combs G.F. (2001). Selenium in global food systems. Brit. J. Nutr..

[18] Kennedy G., Nantel G., Shetty P. (2003). The scourge of ‘hidden hunger’: Global dimensions of micronutrient deficiencies. Food Nutr. Agri..

[19] WHO, FAO Better nutrition, better lives. Proceedings of the Second International Conference on Nutrition (ICN2).

[20] Buturi C.V., Mauro R.P., Fogliano V., Leonardi C., Giuffrida F. (2021). Mineral Biofortification of Vegetables as a Tool to Improve Human Diet. Foods.

[21] Siwela M., Pillay K., Govender L., Lottering S., Mudau F.N., Modi A.T., Mabhaudhi T. (2020). Biofortified crops for combating hidden hunger in South Africa: Availability, acceptability, micronutrient retention and bioavailability. Foods.

[22] Duborská E., Šebesta M., Matulová M., Zvěřina O., Urík M. (2022). Current Strategies for Selenium and Iodine Biofortification in Crop Plants. Nutrients.

[23] Blicharska E., Flieger J., Oszust K., Frąc M., Świeboda R., Kocjan R. (2015). High-resolution continuum source atomic absorption spectrometry with microwave-assisted extraction for the determination of metals in vegetable sprouts. Anal. Lett..

[24] Dunwell J.M. (2014). Transgenic cereals: Current status and future prospects. J. Cereal Sci..

[25] Kumar S., Palve A., Joshi C., Srivastava R.K. (2019). Crop biofortification for iron (Fe), zinc (Zn) and vitamin A with transgenic approaches. Heliyon.

[26] Van Der Straeten D., Bhullar N.K., De Steur H., Gruissem W., MacKenzie D., Pfeiffer W., Bouis H. (2020). Multiplying the efficiency and impact of biofortification through metabolic engineering. Nat. Commun..

[27] Malik K.A., Maqbool A. (2020). Transgenic crops for biofortification. Front. Sustain. Food Syst..

[28] Koç E., Karayiğit B. (2022). Assessment of Biofortification Approaches Used to Improve Micronutrient-Dense Plants That Are a Sustainable Solution to Combat Hidden Hunger. J. Soil Sci. Plant Nutr..

[29] Lorenz K., D’Appolonia B. (1980). Cereal sprouts: Composition, nutritive value, food applications. CRC Cr. Rev. Food Sci..

[30] Park S.A., Grusak M.A., Oh M.M. (2014). Concentrations of minerals and phenolic compounds in three edible sprout species treated with iron-chelates during imbibition. Hortic. Environ. Biotechnol..

[31] Wei Y., Shohag M., Ying F., Yang X., Wu C., Wang Y., Shohag J.I. (2013). Effect of ferrous sulfate fortification in germinated brown rice on seed iron concentration and bioavailability. Food Chem..

[32] Zhu H. (2014). Accumulation and distribution of selenium in different parts and macromolecule of Se-enriched Tartary Buckwheat (*Fagopyrum tataricum* Gaertn.) during germination. Int. Food Res. J..

[33] Liu K., Chen F., Zhao Y., Gu Z., Yang H. (2011). Selenium accumulation in protein fractions during germination of Se-enriched brown rice and molecular weights distribution of Se-containing proteins. Food Chem..

[34] Lazo-Vélez M.A., Avilés-González J., Serna-Saldivar S.O., Temblador-Pérez M.C. (2016). Optimization of wheat sprouting for production of selenium enriched kernels using response surface methodology and desirability function. LWT-Food Sci. Technol..

[35] Krzepiłko A., Zych-Wężyk I., Święciło A., Molas J., Skwaryło-Bednarz B. (2016). Effect of iodine biofortification of lettuce seedlings on their mineral composition and biological quality. J. Elem..

[36] Klepaka T. (2008). Construcion of axial-symmetric polymetric extrudates of complex forms. Polimery.

[37] Arscott S., Goldman I. (2012). Biomass effects and selenium accumulation in sprouts of three vegetable species grown in selenium-enriched conditions. Hortscience.

[38] Zielińska-Dawidziak M., Siger A. (2012). Effect of elevated accumulation of iron in ferritin on the antioxidants content in soybean sprouts. Eur. Food Res. Technol..

[39] Zou T., Xu N., Hu G., Pang J., Xu H. (2014). Biofortification of soybean sprouts with zinc and bioaccessibility of zinc in the sprouts. J. Sci. Food Agric..

[40] Aloo S.O., Ofosu F.K., Kilonzi S.M., Shabbir U., Oh D.H. (2021). Edible Plant Sprouts: Health Benefits, Trends, and Opportunities for Novel Exploration. Nutrients.

[41] He W., Yang H., Gu G. (2008). Effect of Bacteria-mineral water produced from bio-reacted fowl dung on seed germination of wheat (*Triticum aestivum*) and rice (*Oryza sativa* L.). Environ. Progr..

[42] Tripathi D.K., Singh S., Singh S., Mishra S., Chauha D.K., Dubey N.K. (2015). Micronutrients and their diverse role in agricultural crops: Advances and future prospective. Acta Physiol. Plant..

[43] Tripathi D.K., Singh V.P., Prasad S.M., Chauha D.K., Dubey N.K., Rai A.K. (2015). Silicon-mediated alleviation of Cr (VI) toxicity in wheat seedlings as evidenced by chlorophyll florescence, laser induced breakdown spectroscopy and anatomical changes. Ecotoxicol. Environ. Saf..

[44] Sharma P., Jha A.B., Dubey R.S., Pessarakli M. (2012). Reactive oxygen species, oxidative damage, and antioxidative defense mechanism in plants under stressful conditions. J. Bot..

[45] Rupiasih N.N., Vidyasagar P.B. (2016). Effect of UV-C radiation and hypergravity on germination, growth and content of chlorophyll of wheat seedlings. AIP Conf. Proc..

[46] Partap M., Solanki V.A. (2016). Impact of black point incited by Alternaria alternata on wheat trade, seed quality and seed germination. Indian Phytopathol..

[47] Rajput M.A., Pathan M.A., Lodhi A.M., Shah G.S., Khanzada K.A. (2005). Studies on seed-borne fungi of wheat in Sindh Province and their effect on seed germination. Pak. J. Bot..

[48] Noble R.E. (2002). Effects of UV-irradiation on seed germination. Sci. Total Environ..

[49] Kuzdraliński A., Kot A., Szczerba H., Nowak M., Muszyńska M. (2017). A review of conventional PCR assays for the detection of selected phytopathogens of wheat. J Mol. Microbiol. Biotechnol..

[50] Singh V.K., Devi A., Pathania S., Kumar V., Tripathi D.K., Sharma S., Chauhan D.K., Singh V.K., Zorba V. (2017). Spectroscopic investigation of wheat grains (*Triticum aestivum*) infected by wheat seed gall nematodes (*Anguina tritici*). Biocat. Agri. Biotech..

[51] Badridze G., Kacharava N., Chkhubianishvili E., Rapava L., Kikvidze M., Chanishvili S., Shakarishvili N., Mazanishvili L., Chigladze L. (2016). Effect of UV radiation and artificial acid rain on productivity of wheat. Russ. J. Ecol..

[52] Thomas D.T.T., Puthur J.T. (2017). UV radiation priming: A means of amplifying the inherent potential for abiotic stress tolerance in crop plants. Environ. Exp. Bot..

[53] Brown J., Lu T., Stevens C., Khan V., Lu J., Wilson C., Collins D., Wilson M., Igwegbe E., Chalutz E. (2001). The effect of low dose ultraviolet light-C seed treatment on induced resistance in cabbage to black rot (*Xanthomonas campestris* pv. campestris). Crop Prot..

[54] Kacharava N., Chanishvili S., Badridze G., Chkhubianishvili E., Janukashvili N. (2009). Effect of seed irradiationon the content of antioxidants in leaves of kidney bean, cabbage and beet cultivars. Aust. J. Crop. Sci..

[55] Ouhibi C., Attia H., Rebah F., Msilini N., Chebbi M., Aarrouf J., Urban L., Lachaal M. (2014). Salt stress mitigation by seed priming with UV-C in lettuce plants, growth, antioxidant activity and phenolic compounds. Plant Phys. Biochem..

[56] Shetta N.D., Areaf I.M. (2009). Impact of ultraviolet-c radiation on seed germination and chlorophyll concentration of some woody trees grown in Saudi Arabia. J. Agric. Food Env. Sci..

[57] Brazaitytė A., Viršilė A., Jankauskienė J., Sakalauskienė S., Samuolienė G., Sirtautas R., Novičkovas A., Dabašinskas L., Miliauskienė J., Vaštakaitė V. (2015). Effect of supplemental UV-A irradiation in solid-state lighting on the growth and phytochemical content of microgreens. Int. Agrophysics.

[58] Shukla U.C., Kakkar P. (2002). Effect of dual stress of ultraviolet-B radiation and cadmium on nutrient uptake of wheat seedlings. Commun. Soil Sci. Plant..

[59] Ilyas M., Khan M.J., Murad Z., Satti S.Z., Ullah A. (2022). Biofortification of Iron in Wheat Varieties Using Different Methods of Application. Gesunde Pflanz..

[60] Dhaliwal S.S., Sadana U.S., Manchanda J.S., Dhadli H. (2009). Biofortification of wheat grains with zinc and iron in Typic Ustochrept soils of Punjab. Indian J. Fertil..

[61] Dhaliwal S.S., Sharma V., Shukla A.K., Verma V., Kaur M., Shivay Y.S., Nisar S., Gaber A., Brestic M., Barek V. (2022). Biofortification—A Frontier Novel Approach to Enrich Micronutrients in Field Crops to Encounter the Nutritional Security. Molecules.

[62] Wu C.-Y., Lu L.-L., Yang X.-E., Feng Y., Wei Y.-Y., Hao H.-L., Stoffella P.J., He Z.-L. (2010). Uptake, translocation, and remobilization of zinc absorbed at different growth stages by rice genotypes of different Zn densities. J. Agric. Food Chem..

[63] Cakmak I. (2008). Enrichment of cereal grains with zinc: Agronomic or genetic biofortification?. Plant Soil..

[64] Phattarakul N., Rerkasem B., Li L.J., Wu L.H., Zou C.Q., Ram H., Sohu V.S., Kang B.S., Surek H., Kalayci M. (2012). Biofortification of rice grain with zinc through zinc fertilization in different countries. Plant Soil..

[65] Cakmak I., Kalayci M., Kaya Y., Torun A.A., Aydin N., Wang Y., Arisoy Z., Erdem H., Yazici A., Gokmen O. (2010). Biofortification and localization of zinc in wheat grain. J. Agric. Food Chem..

[66] Velu G., Ortiz-Monasterio I., Cakmak I., Hao Y., Singh R.P. (2014). Biofortification strategies to increase grain zinc and iron concentrations in wheat. J. Cereal Sci..

[67] Guttieri M.J., Peterson K.M., Souza E.J. (2006). Agronomic performance of low phytic acid wheat. Crop. Sci..

[68] Liu Z.H., Wang H.Y., Zhang G.P., Chen P.D., Liu D.J. (2006). Genotypic and spike positional difference in grain phytase activity, phytate, inorganic phosphorus, iron and zinc contents in wheat (*Triticum aestivum* L.). J. Cereal Sci..

[69] Bouis H.E., Welch R.M. (2010). Biofortification—A sustainable agricultural strategy for reducing micronutrient malnutrition in the Global South. Crop. Sci..

[70] Courtois D., Kasternmayer P., Clough J., Vigo M., Sabatier M., Arnaud M.J. (2003). Magnesium enrichment and distribution in plants. Isot. Environ. Health Stud..

[71] ISTA International rules for seed testing. Proceedings of the Ordinary General Meeting 2016.

